# Exploring the impact of stimulant medications on weight in children with attention deficit hyperactivity disorder in Dubai, United Arab Emirates

**DOI:** 10.3389/fpsyt.2024.1392846

**Published:** 2024-10-16

**Authors:** Faten Al Eid, Ammar Albanna, Jessie Joseph, Sami Talo, Lakshmanan Jeyaseelan, Meshal A. Sultan

**Affiliations:** ^1^ College of Medicine, Mohammed Bin Rashid University of Medicine and Health Sciences, Dubai Health, Dubai, United Arab Emirates; ^2^ Mental Health Centre of Excellence, Al Jalila Children’s Specialty Hospital, Dubai Health, Dubai, United Arab Emirates; ^3^ Al Amal Psychiatric Hospital, Emirates Health Services, Dubai, United Arab Emirates

**Keywords:** psychostimulants, weight, children, adolescents, attention deficit hyperactivity disorder (ADHD)

## Abstract

**Objective:**

Attention deficit hyperactivity disorder (ADHD), prevalent in 5% of children worldwide, impacts academic performance and often coexists with psychiatric disorders. Psychostimulant medications are primary treatments for ADHD, enhancing dopamine to reduce symptoms. However, dopamine increase may cause appetite loss. This pioneering study in the United Arab Emirates (UAE) explores psychostimulant effects on weight in children diagnosed with ADHD, aiming to uncover unique regional characteristics and contributing factors to weight changes.

**Methods:**

This retrospective cohort study assessed data from electronic medical records from 2017 to 2022, aiming to assess the impact of psychostimulants on weight in children aged 6–18 years. Inclusion criteria covered psychostimulant-treated and untreated patients with ADHD. Statistical analysis, involving longitudinal data methods aimed to demonstrate significant weight differences.

**Results:**

Data from 107 pediatric patients diagnosed with ADHD were analyzed, with 86 meeting inclusion criteria. Most patients were male (80.2%). ADHD presentations varied, and methylphenidate immediate release was the most prescribed stimulant medication. Patients experienced initial weight loss followed by overall gain over 12 months. Coexisting conditions, maternal factors, family history, and correlations with autism spectrum disorder were explored.

**Conclusion:**

This study provides valuable insights into the effects of psychostimulant medications on the weight of children and adolescents diagnosed with ADHD in the UAE. It suggests avenues for future research, emphasizing extended follow-ups to understand long-term psychostimulant effects, nuanced examinations of age and gender, and exploring interactions with comorbidities. Despite limitations, the research provides insights into ADHD medication effects, guiding personalized treatment approaches for pediatric populations.

## 1 Introduction

Attention deficit hyperactivity disorder (ADHD) is considered among the most prevalent neurodevelopmental disorders among children and adolescents. However, the prevalence of ADHD can vary significantly depending on the type of study and the region. For example, a meta-analysis reported a global prevalence of 7.2%, among children and 5.6% among adolescents, with substantial variability across countries due to differences in study design, diagnostic tools, and population demographics (1)​​​​. In Europe, a systematic review found that the prevalence rates in children and adolescents ranged widely between studies from 1.3% to 21.8% (2). Another study from Slovenia reported a gradual increase in the annual incidence rate of ADHD in children and adolescents from 0.032% in 1997 to 0.082% in 2012 (3). Studies conducted in Africa estimated a prevalence of 7.47%, with significant variation between countries, ranging from 1.49% in Ethiopia to 11.75% in Uganda​​ (4).

Presentation of ADHD includes hyperactivity/impulsivity, inattention or a combination of both (5). ADHD can impair academic performance as well as interpersonal interactions (6). Moreover, children and adolescents with ADHD have a higher rate of co-morbid psychiatric disorders including anxiety disorders, depressive disorders, conduct disorder, and learning problems (7). Therefore, setting a management that is tailored to the needs of the patient is crucial in order to achieve a better prognosis.

Pharmacoepidemiological studies have provided valuable insights into the patterns of ADHD medication use and their impacts. For instance, a 10-year study in Germany showed that the prescription rates for ADHD medications, particularly methylphenidate, have varied significantly over time, with notable increases in prescriptions for older adolescents and adults (8)​​. This trend reflects changes in clinical practice and possibly greater recognition of ADHD in older age groups. Additionally, research has shown that ADHD medications can reduce the risk of substance use disorders and other comorbid conditions, emphasizing their importance in comprehensive ADHD management​​​​ (9, 10).

Psychostimulants in their different formulations, amphetamine-based salts and methylphenidate medications, are the first line treatment options for ADHD. They are known for being effective, well tolerated with minor side effects. Moreover, stimulants have been shown to be effective and well tolerated in both specialized and primary care clinical settings in the UAE (11). The mechanism in which psychostimulants exert their effect is thought to be by increasing both norepinephrine and dopamine which in return improves attentiveness and reduces hyperactivity and impulsivity (12). However, an increase in the latter neurotransmitter is thought to be responsible for appetite loss through stimulation of the disgust sensation via the activation of the insular lobe (12).

Methylphenidate, a commonly prescribed psychostimulant for ADHD, exerts its effects through a well-defined pharmacodynamic mechanism. It functions primarily by blocking the reuptake of dopamine and norepinephrine into the presynaptic neuron, thereby increasing the availability of these neurotransmitters in the synaptic cleft (12). This blockade is achieved by inhibiting the dopamine transporter and the norepinephrine transporter, leading to an enhanced neurotransmitter release into the extraneuronal space. The increased levels of dopamine and norepinephrine in the synaptic cleft result in improved neurotransmission and modulation of attention and behavior, which are often impaired in individuals with ADHD. This mechanism not only augments synaptic concentrations of these monoamines but also amplifies their postsynaptic receptor activation, thereby enhancing attentiveness and reducing hyperactive and impulsive behaviors (13).

The most common side effects of psychostimulants include headaches, insomnia, and appetite loss (5). Methylphenidate poses an anorexigenic effect which results in appetite suppression and, hence, weight loss. However, this effect is prominent during the first 3–6 months after the initiation of a psychostimulant (12). Moreover, most subjects receiving psychostimulants tend to have similar weight curve to those not receiving psychostimulants after few years (12).

In one study, pre-treatment weight adjusted for gender, age, and height was found to be a significant predictor of weight loss in children with ADHD treated with psychostimulants including methylphenidate or dextroamphetamine (14). In another study, family history of a specific response and side effect profile (e.g., weight loss) with use of a medication such as methylphenidate had a significant correlation with the probability of developing similar response and side effects in another family member treated with the same medication (15). Moreover, weight loss and appetite suppression were not limited to drug use but were rather also associated with drug discontinuation, as per the literature (16). Additionally, it was shown in multiple studies that weight loss with amphetamine and dextroamphetamine is more profound compared to methylphenidate (17, 18).

Exploring the magnitude of the impact of different formulations of psychostimulants on growth parameters in children with ADHD is essential; it will improve our understanding of the effect of these medications on changes in weight. Moreover, exploring this area may reveal certain characteristics that are unique to the population in the Arab region and might be different compared to other regions in the world. In fact, this study is the first to be done on the effect of psychostimulants on weight in children and adolescents diagnosed with ADHD in the United Arab Emirates (UAE) and in the Middle East. The main aim of this study is to evaluate the effect of psychostimulants on the weight of children and adolescents with ADHD. Furthermore, the study aims to explore possible contributing factors to the progression of weight over time.

## 2 Methods

### 2.1 Study design, setting, and participants

This is a retrospective cohort study. Data collection was based on information obtained from the electronic medical records database of patients who presented at mental health services at Al Jalila Children’s Specialty Hospital in Dubai, UAE, through the years 2017–2022.

In terms of the inclusion criteria, pediatric patients aged 6–18 years diagnosed with ADHD, evaluated at mental health services, taking psychostimulants (mixed amphetamine salts, dextroamphetamine, or methylphenidate) for a period of at least 6 months, as well as patients with ADHD not treated with psychostimulant medications, were eligible to be in this study. The exclusion criteria consisted of having a comorbid diagnosis of eating disorder, or major depressive disorder, or taking any other medication that may impact growth parameters.

The diagnosis of ADHD and the identification of comorbidities were conducted based on clinical assessments by qualified child and adolescent psychiatrists using the criteria outlined in the Diagnostic and Statistical Manual of Mental Disorders, Fifth Edition (DSM-5) (19).

### 2.2 Variables

Variables obtained from the patients database for this study included demographics: age and gender; primary diagnosis of ADHD; specifiers of the disorder which were categorized into three groups: predominantly inattentive, predominantly hyperactive/impulsive and combined presentations; the type of prescribed psychostimulant medications which included methylphenidate (MPH) immediate release, methylphenidate osmotic-release oral system (MPH OROS), amphetamine mixed salts (AMPs) immediate release, amphetamine mixed salts extended release, and Lisdexamfetamine. Lisdexamfetamine and AMPs extended release were further categorized under amphetamine-based extended release medications. The absolute weights of the participants were converted to age- and sex-adjusted weight-for-age *z*-scores to standardize the weight data across the diverse age range of the study population. The weight-for-age *z*-scores were calculated using reference data from the World Health Organization growth standards for children aged 5 years and below and the Centers for Disease Control and Prevention Growth Charts for those aged above 5 years. This standardization process allows for a more accurate comparison of weight changes relative to expected growth patterns based on the participants’ age and sex (20, 21).

The analyzed co-morbid conditions included intellectual disability (ID), language disorder, motor disorder, learning disorder, anxiety disorder, oppositional defiant disorder (ODD), conduct disorder, tic disorder, obsessive compulsive disorder (OCD), post-traumatic stress disorder (PTSD), sleep problem, sleep disorder, epilepsy, enuresis, and autism spectrum disorder (ASD). Additionally, factors related to the patient’s mother’s health during pregnancy and certain pregnancy characteristics were analyzed and included gestational diabetes, psychosocial stress, pre/post-partum depression in the mother, smoking, alcohol, drugs, delivery method, birth weight (in kg). Disorders in the child that were analyzed included heart disease, thyroid disease, head injury, and asthma. Relevant family history in first- and second-degree relatives was also analyzed and included ADHD, ASD, ID, language disorder, motor disorder, ODD, anxiety disorder, MDD, Fragile X syndrome, epilepsy, substance use disorder, suicide, bipolar disorder, schizophrenia, eating disorder, and consanguinity.

The rationale for assessing maternal factors and family history in this study is based on the understanding that both prenatal influences and genetic predispositions can significantly impact the health outcomes of children, including those diagnosed with ADHD. Maternal factors such as gestational diabetes, psychosocial stress, and maternal mental health can affect fetal development and have been associated with various neurodevelopmental outcomes (22, 23). Specifically, maternal stress and depression during pregnancy have been linked to alterations in fetal brain development and an increased risk of ADHD and other behavioral disorders in offspring (24). Similarly, family history of ADHD and other psychiatric conditions can indicate a genetic predisposition to these disorders (25). Studies have shown that children with a family history of ADHD are at a higher risk of developing the disorder themselves, and this genetic susceptibility can also influence their response to treatment and associated health outcomes, such as weight changes (26). By including these maternal and familial factors, we aim to explore both prenatal and genetic influences on the child’s health outcomes, which could indirectly impact weight changes observed during psychostimulant treatment.

### 2.3 Data sources/measurement

Using the data collection tool generated by the team, we extracted relevant data from the electronic medical record database of a representative sample population of children and adolescents diagnosed with ADHD at Al Jalila Children’s Specialty Hospital, Dubai, UAE. The data collection tool obtained various demographic information, which included age, gender, as well as information relating to diagnoses, duration of psychostimulants uses, weight before receiving psychostimulants and weight change after receiving psychostimulants, height before receiving psychostimulants and height change after receiving psychostimulants, history of neurodevelopmental disorders, and family history of members treated with stimulant medication. The clinical information was based on clinical assessment and psychometric analysis findings from diagnosed patients in the outpatient setting during the defined time for the study.

### 2.4 Statistical methods

Sample size calculation was based on findings from the study by Swanson et al. (12). For the weight difference between the two groups: at 14 months, the mean (SD) of weight for the “No Medication group” was 0.617 (0.915) while this was 0.113 (1.14) in the “New medication” group. In order to show that this difference is statistically significant with alpha error and power at 5% and 80%, respectively, we required a sample size of 50 subjects in each group. Therefore, we aimed for assessing 100 subjects for the purpose of our study.

Study variables that were categorical such as gender and family history were presented with numbers and percentages. The variables that were continuous such as age, height, and weight were presented with mean and SD. The weight data were further standardized by converting absolute weight values to weight-for-age *z*-scores to account for the broad age range of participants. This adjustment allows for a more accurate comparison of weight changes over time. The data were examined for extreme values using frequency distribution and box plots. The outcome variables of weight were measured at the baseline and monthly for 12 months (repeated measures). As the observations (weight) are correlated over time, longitudinal data analyses [Generalized Estimating Equation (GEE)] with normal distribution and with exchangeable correlation matrix was done. Over time effect and intervention effects were tested after adjusting for the confounders such as age and gender. Actual *p*-values were presented. However, *p*-value up to 0.05 were interpreted as clinically significant associations or values. Data were analyzed using the Statistical Package for the Social Sciences and Statistical software for data science (STATA) software.

## 3 Results

### 3.1 Characteristics of participants

**Figure 1:**
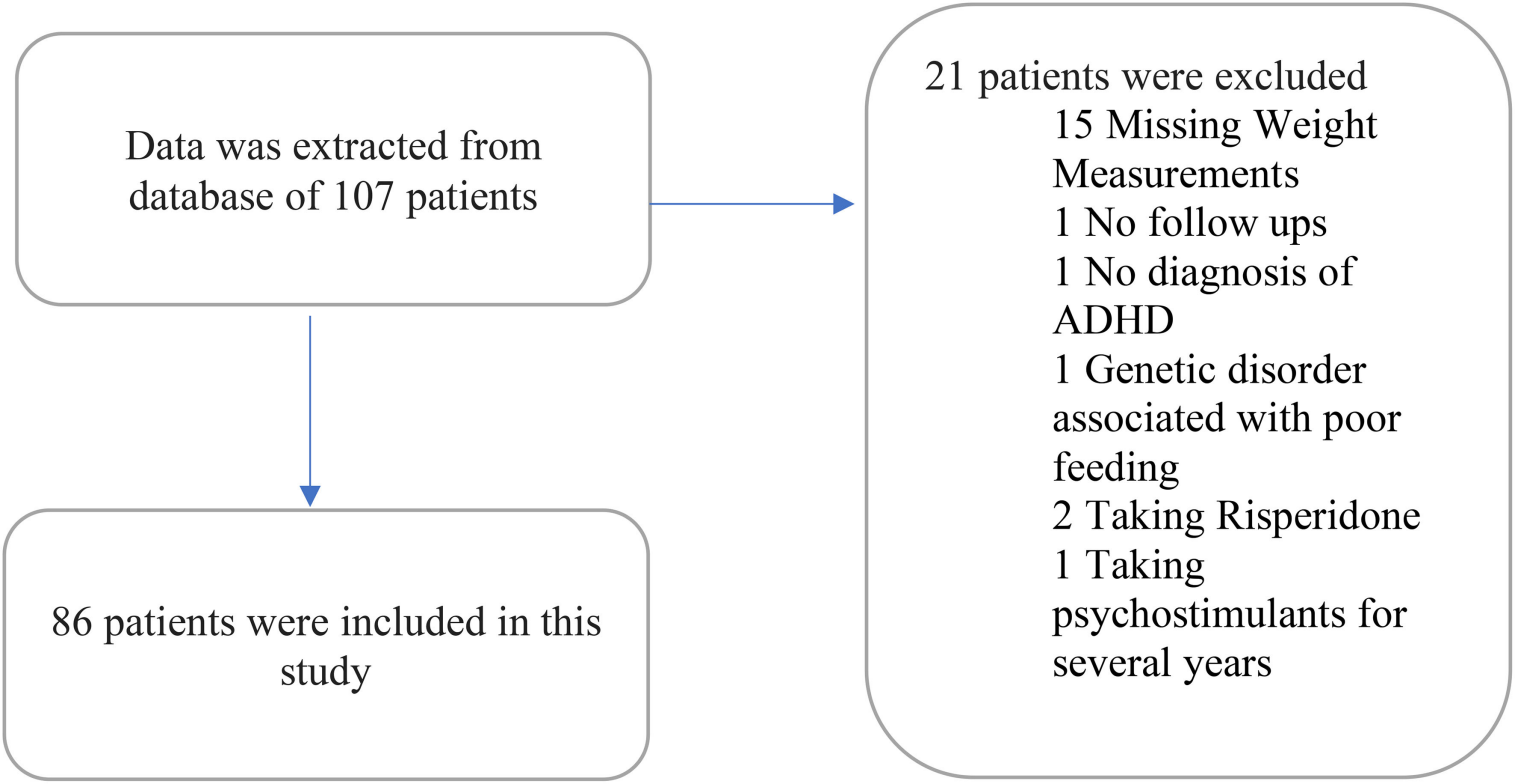
Flowchart of included patients.

Data were extracted from the electronic medical record database for 107 patients, and data from the 86 patients who met the inclusion criteria were analyzed. Twenty-one patients were excluded due to the following reasons: 15 patients were excluded due to the missing data about their weight measurements; one patient due to the lack of any follow-up visits to the psychiatry service as he was referred for a consultation; one patient due to the absence of a formal diagnosis of ADHD; one patient due to being diagnosed with a genetic disorder that results in poor feeding; two patients due to taking a psychotropic that impacts weight (risperidone); one patient due to being on a psychostimulant for years prior to seeking our psychiatric service. Out of the 86 included patients (**Figure 1**), 78 were on psychostimulant medications and 8 patients were not on any medication. Patients on medications were as per the following: 37 (43.5%) on methylphenidate immediate release; 27 (31.4%) on methylphenidate osmotic-release oral system (OROS); 3 (3.6%) on amphetamine mixed salts immediate release; 11 (13.1%) on amphetamine-based extended release (lisdexamfetamine or amphetamine mixed salts extended release).

In terms of the gender of the included sample (*n* = 86), 80.2% of the children and adolescents in this study were male, and 19.8% were female. The mean (*SD*) age in years of the patients was 12 (3). The mean (*SD*) age in years of the parents was 32 (7) for mothers (*n* = 45) and 37 (9) for fathers (*n* = 45). Among parents (*n* = 45) in the data that were analyzed, consanguinity was identified in nine parents.

ADHD presentations were categorized into three groups: predominantly inattentive, predominantly hyperactive/impulsive, and combined presentations. 10.8% and 4.8% were diagnosed as having a predominantly inattentive and predominantly hyperactive/impulsive presentation, respectively. 45.8% of patients were diagnosed with the combined presentation. In 21.7% of the patient sample, the ADHD subtype was not confirmed.

### 3.2 Co-occurring disorders and potential risk factors

Furthermore, various disorders co-occurring in these patients with ADHD were analyzed. Intellectual disability (ID) was found in 18.6% of patients, language disorder in 26.6%, motor disorder in 3.9%, learning disorder in 25.7%, anxiety disorder in 16.4%, oppositional defiant disorder (ODD) in 10.8%, conduct disorder in 9.5%, tic disorders in 5.4%, obsessive-compulsive disorder (OCD) in 4.3%, PTSD in 1.4%, sleep problems in 22.2%, epilepsy in 12.2%, enuresis in 6.8%, ASD in 38.8%, heart disease in 1.3%, head injury in 6.7%, and asthma in 10.3%. None of the patients were found to have fragile X syndrome, sleep disorders, or thyroid disease.

As for maternal pregnancy complications, gestational diabetes was identified in 17.8% (*n* = 73), psychosocial stress in 19.5% (*n* = 77), and pre-/post-partum depression in 12.5% (*n* = 72) of the patients’ mothers. Additionally, the mode of delivery, birth weight of patients, and maternal behavioral factors during pregnancy such as smoking, alcohol consumption, and drug use were also assessed. 64.2% (*n* = 43) of patients were delivered via normal vaginal delivery, and 34.3% (*n* = 23) were delivered via cesarean section. The mean (SD) birth weight was identified as 3.11 kg (0.61), *n* = 65. As for behavioral factors, 1.6% (*n* = 62) of the mothers consumed alcohol during their pregnancy, and none (*n* = 62) of them smoked or used substances.

Family history of psychiatric disorders was another area that was analyzed in this study. Family history of ADHD in a first degree relative was 9.7% (*n* = 62) and in a second degree relative was 12.9% (*n* = 62). Family history of ASD in a first degree relative was 7.7% (*n* = 65) and in a second degree relative was 17.5% (*n* = 63). Family history of an anxiety disorder in a first degree relative was 9.8% (*n* = 61) and in a second degree relative was 3.3% (*n* = 60). Family history of major depressive disorder in a first degree relative was 13.3% (*n* = 60) and in a second degree relative was 3.3% (*n* = 60). Family history of language disorder in a first degree relative was 3.2% (*n* = 62). Family history in a first degree relative was between 1 and 2% in each the following disorders: intellectual disability, epilepsy, motor disorder, substance use disorder, bipolar disorder, and eating disorders. Family history in a first degree relative was absent for oppositional defiant disorder, schizophrenia, and fragile X.

### 3.3 Impact of stimulant medications on weight

This study investigated the prescription of stimulant medications, which included MPH immediate-release, MPH OROS, AMP immediate release, and Amphetamine-based extended release. MPH immediate release was given to 43.5% (*n* = 37) of patients, while 31.4% (*n* = 27) of patients received MPH OROS, 3.6% (*n* = 3) received AMP immediate release, and 13.1% (*n* = 11) received Amphetamine-based extended release. Assessment of whether patients were switched to long-acting formulations and, if so, when was performed. 20.6% of patients were shifted to long-acting medications, of which 6.4% and 5.1% were shifted in the first and second months, respectively. 2.6% of individuals were started on extended-release formulations in the third and sixth months, and 1.3% in the fourth and seventh months. None of the patients were moved to long-acting formulations between the eighth and 12th month after initiation of short-acting formulation.

**Figure 2:**
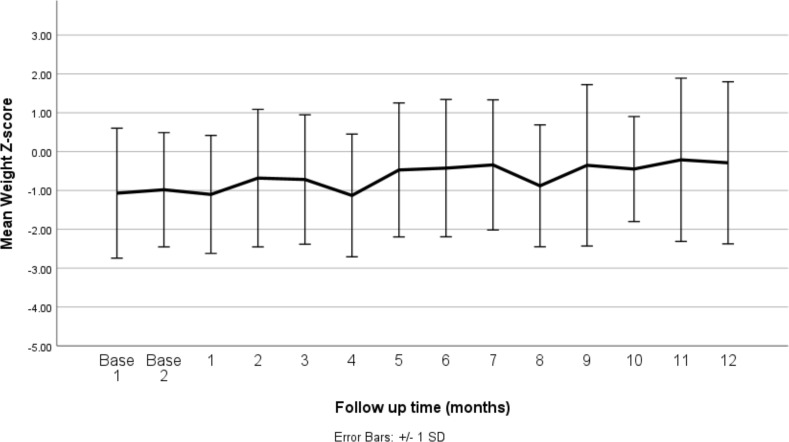
Mean weight *z*-score at baseline (before medication) and changes by months of follow up (after medication). SD, standard deviation.

**Figure 2** presents the mean weight-for-age *z*-scores at baseline and over the 12-month follow-up period after the initiation of stimulant medication. The baseline *z*-scores indicate that, on average, patients had slightly lower than expected weights for their age and sex, with mean *z*-scores around −1.0 at the start of the study. Over the course of the follow-up, the mean weight-for-age *z*-scores remained relatively stable, showing slight fluctuations but generally staying within the range of −1.0 to 0.0. This suggests that while the stimulant medications had an initial impact, leading to minor weight reductions in some patients, the overall effect on weight normalized over time, as indicated by the stabilization of the *z*-scores. The error bars represent the standard error, reflecting the variability in weight-for-age *z*-scores across the patient population. The relatively consistent mean *z*-scores across the follow-up period indicate that the stimulant medications did not result in significant deviations from the expected growth patterns in this cohort.

**Table 1** presents the mean weight-for-age *z*-scores at baseline and at each follow-up time point over the 12-month period following the initiation of stimulant medication. At baseline, the mean *z*-scores were slightly below the expected norm, with values of −1.07 and −0.98, indicating that, on average, patients were underweight relative to their age and sex. Following the initiation of medication, the mean weight-for-age *z*-scores initially fluctuated, with a notable decrease to −1.13 at 4 months, reflecting a further decline in weight relative to the expected growth curve. However, from the fifth month onward, the *z*-scores began to recover, showing a gradual increase. By the 12th month, the mean *z*-score improved to −0.29, suggesting a partial recovery towards the expected weight range for age and sex. The standard deviations remained relatively consistent throughout the period, indicating a similar level of variability in weight-for-age *z*-scores across the study population.

**Table 1:** Mean and standard deviation of weight *z*-score at baseline and after medication.

Weight *z*-score	Mean	Standard deviation	*n*
WZ1	−1.07	1.67	51
WZ2	−0.98	1.47	30
WZM1	−1.10	1.52	25
WZM2	−0.68	1.77	21
WZM3	−0.72	1.67	14
WZM4	−1.13	1.58	19
WZM5	−0.47	1.72	12
WZM6	−0.43	1.77	12
WZM7	−0.34	1.68	8
WZM8	−0.88	1.57	11
WZM9	−0.35	2.08	13
WZM10	−0.45	1.35	12
WZM11	−0.21	2.10	11
WZM12	−0.29	2.09	11

WZ1, first weight z-score measurement prior to medications; WZ2, second weight z-score measurement prior to medications; WZM1, weight z-score at 1 month on medication; WZM2, weight z-score at 2 months on medication; WZM3, weight z-score at 3 months on medication; WZM4, weight z-score at 4 months on medication; WZM5, weight z-score at five months on medication; WZM6, weight z-score at 6 months on medication; WZM7, weight z-score at 7 months on medication; WZM8, weight z-score at 8 months on medication; WZM9, weight z-score at nine months on medication; WZM10, weight z-score at 10 months on medication; WZM11, weight z-score at 11 months on medication; WZM12, weight z-score at 12 months on medication; n, number of patients.

**Figure 3:**
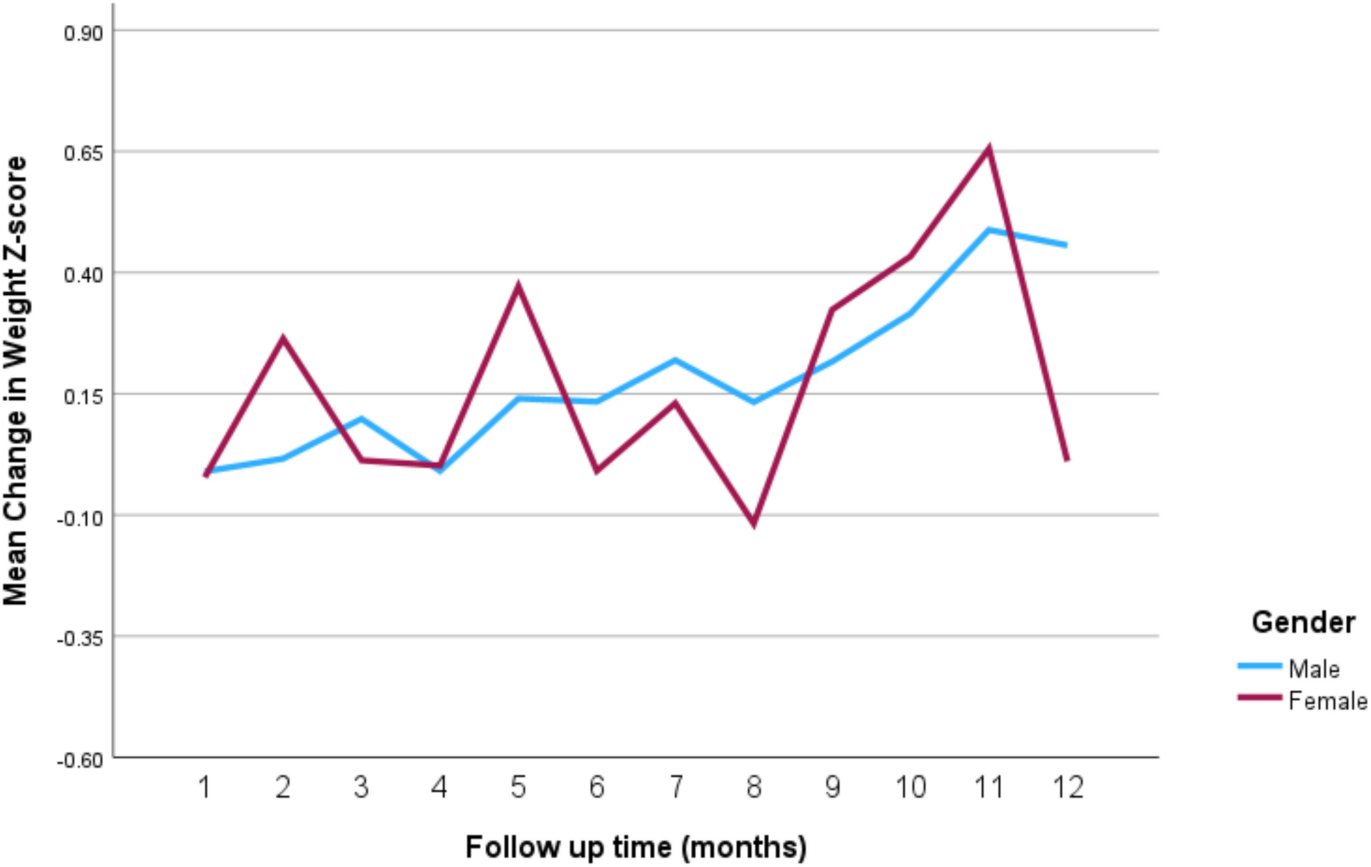
Change in mean weight *z*-score over time according to gender. Standard errors were not provided as the female gender number are small and therefore the error bars are very high.

**Figure 3** represents the mean weight *z*-score of patients distinguished by gender and plotted against time. However, the number of female patients followed up over time was very small. **Table 2** presents the results of the longitudinal data analysis using GEE to assess the impact of stimulant medications on weight-for-age *z*-scores, taking into account age, gender, and time. The analysis revealed a significant positive association between age and weight-for-age *z*-scores (regression coefficient = 0.433, *p* < 0.001), indicating that as patients age, their weight-for-age *z*-scores tend to increase, suggesting an improvement in weight relative to expected growth patterns. The gender variable (female) showed a positive coefficient (0.312), but this was not statistically significant (*p* = 0.523), implying no significant difference in the impact of stimulant medications on weight-for-age *z*-scores between male and female patients. Additionally, the time variable showed a significant positive association with weight-for-age *z*-scores (regression coefficient = 0.038, *p* < 0.001), indicating that over the follow-up period, patients’ weight-for-age *z*-scores generally improved.

**Table 2:** Longitudinal data analysis of impact of stimulant medications on weight *z*-score according to age, gender, and time.

Parameter	Regression coefficient	95% Wald confidence interval		
		Lower	Upper	*P*-value
(Intercept)	−6.644	−7.965	−5.323	< 0.001
Age	0.433	0.323	0.542	< 0.001
Female	0.312	−0.645	1.269	0.523
Time in months	0.038	0.023	0.054	< 0.001

**Figure 4:**
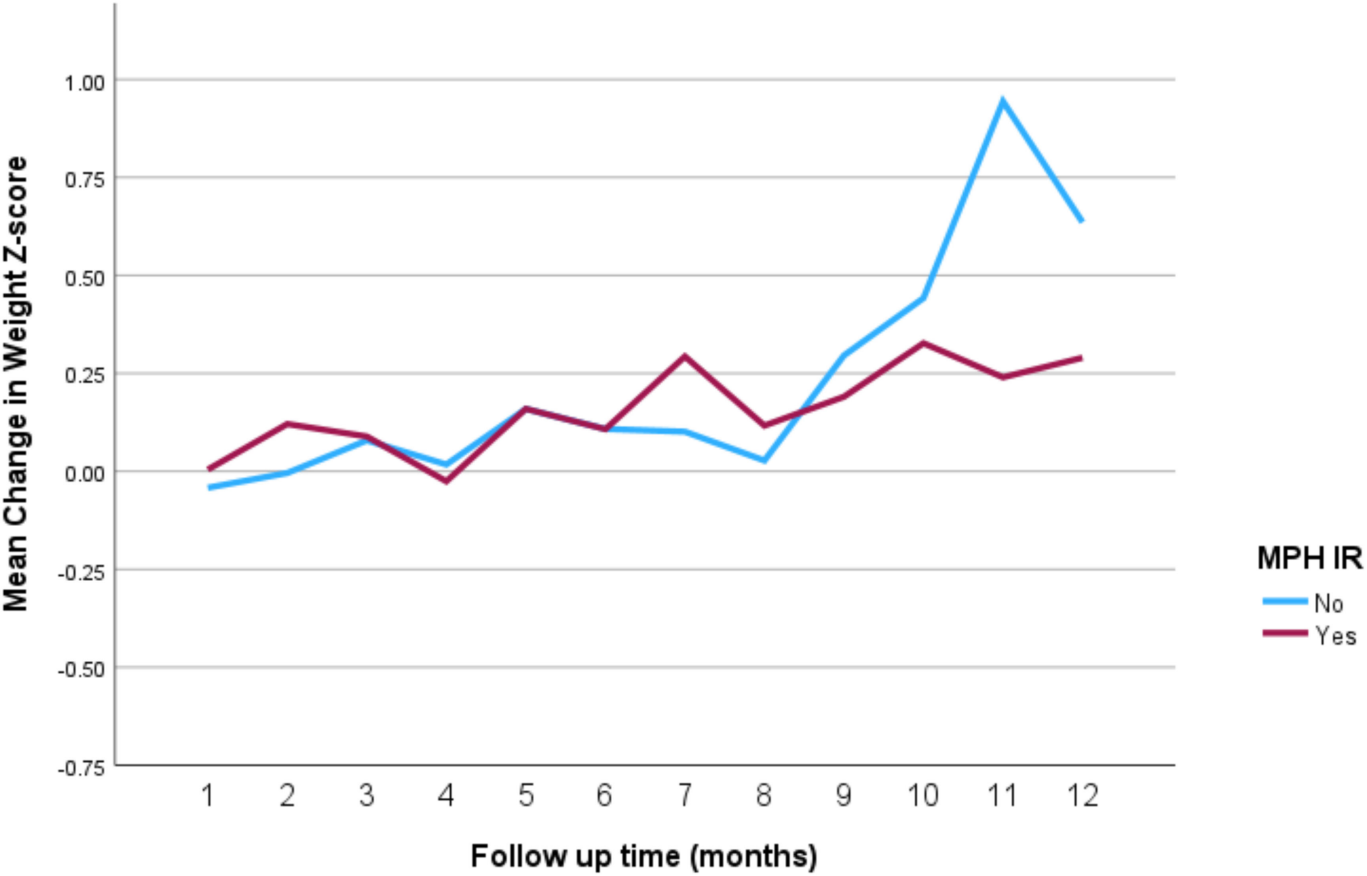
Impact of methylphenidate immediate release on weight over a 12-month period. MPH IR, methylphenidate immediate release.

**Figure 5:**
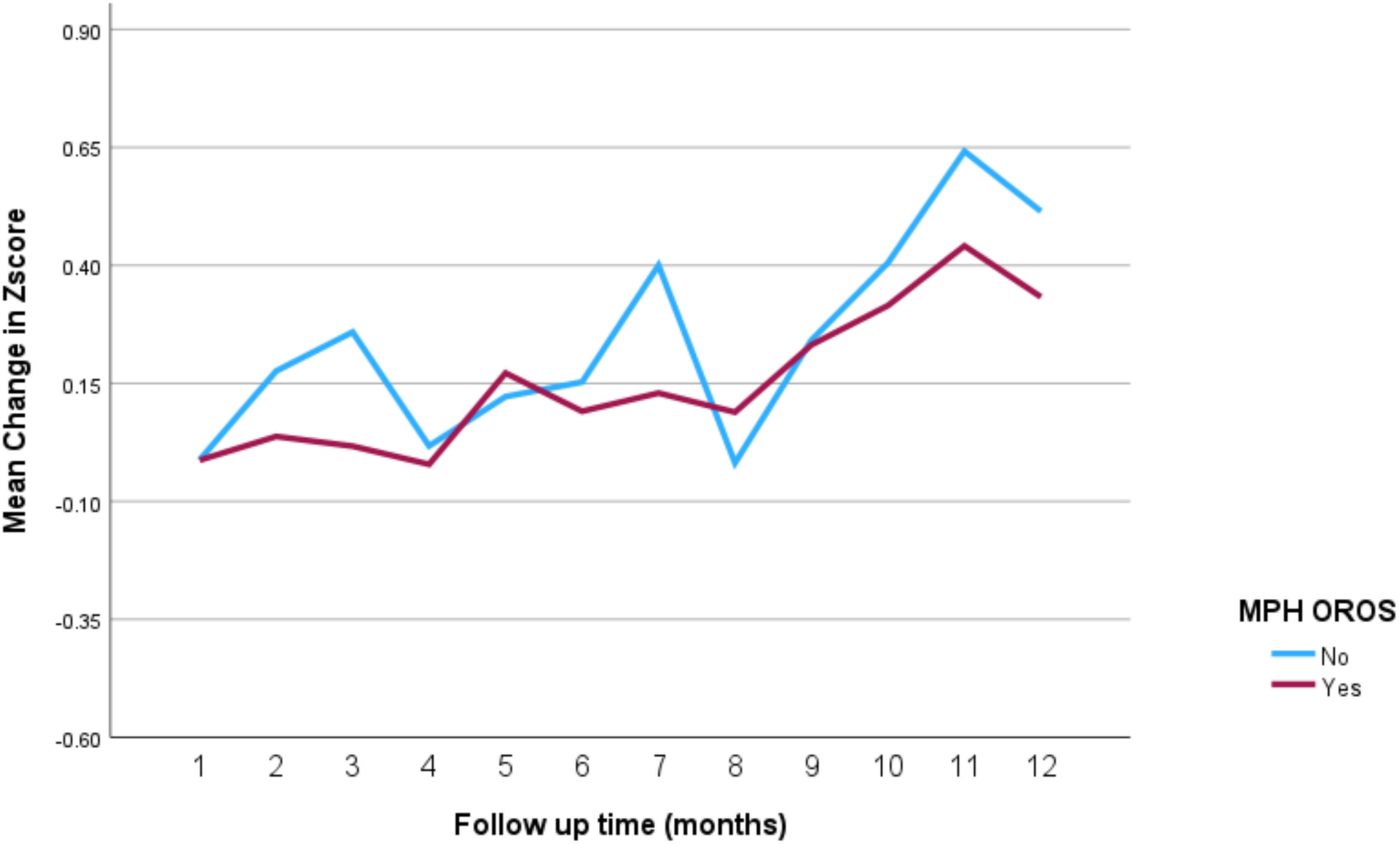
Impact of methylphenidate osmotic release oral system on weight over a 12-month period. MPH OROS, methylphenidate osmotic release oral system.

**Figure 6:**
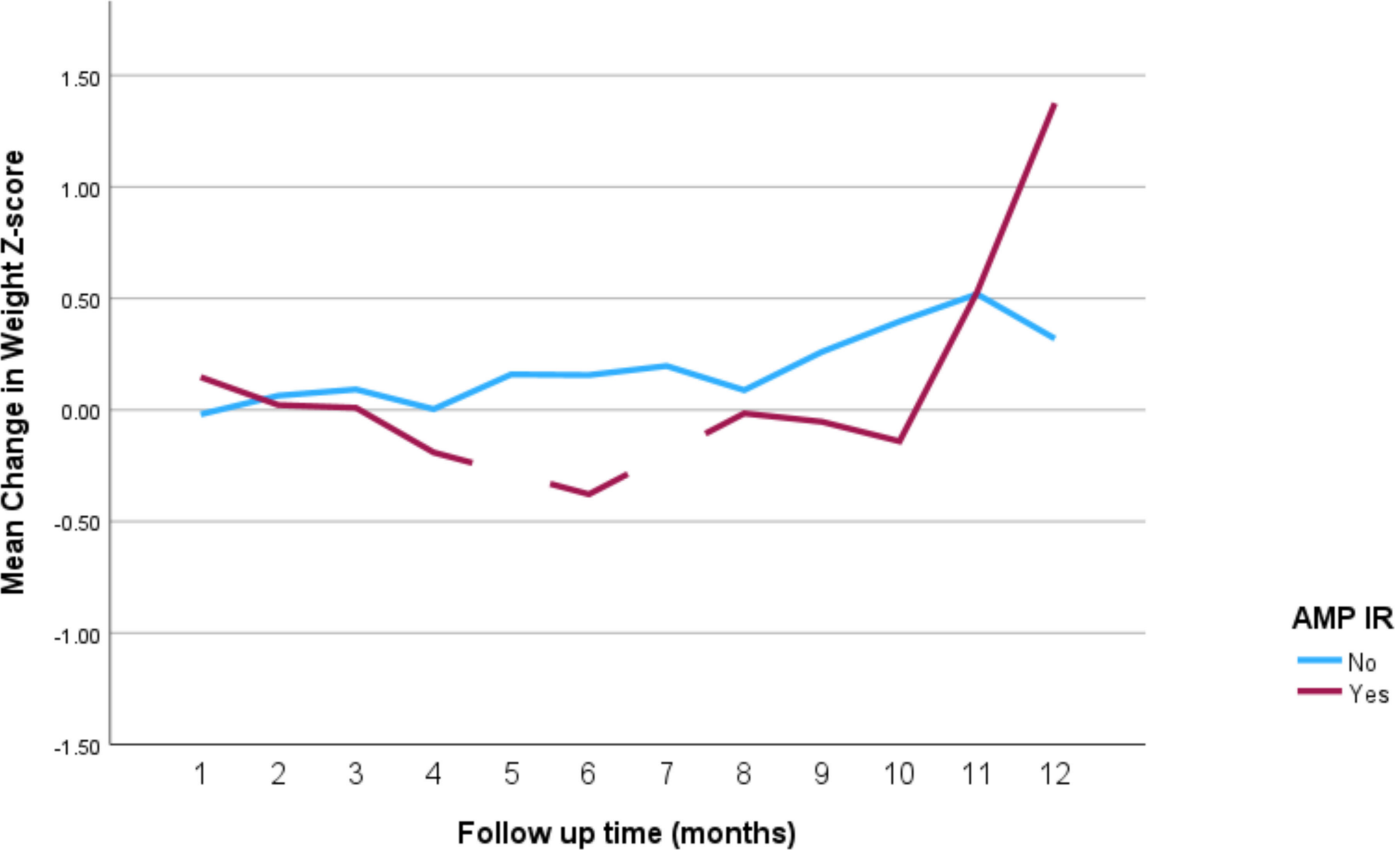
Impact of amphetamine mixed salts immediate release on weight over a 12-month period. AMP IR, amphetamine mixed salts immediate release.

**Figure 7:**
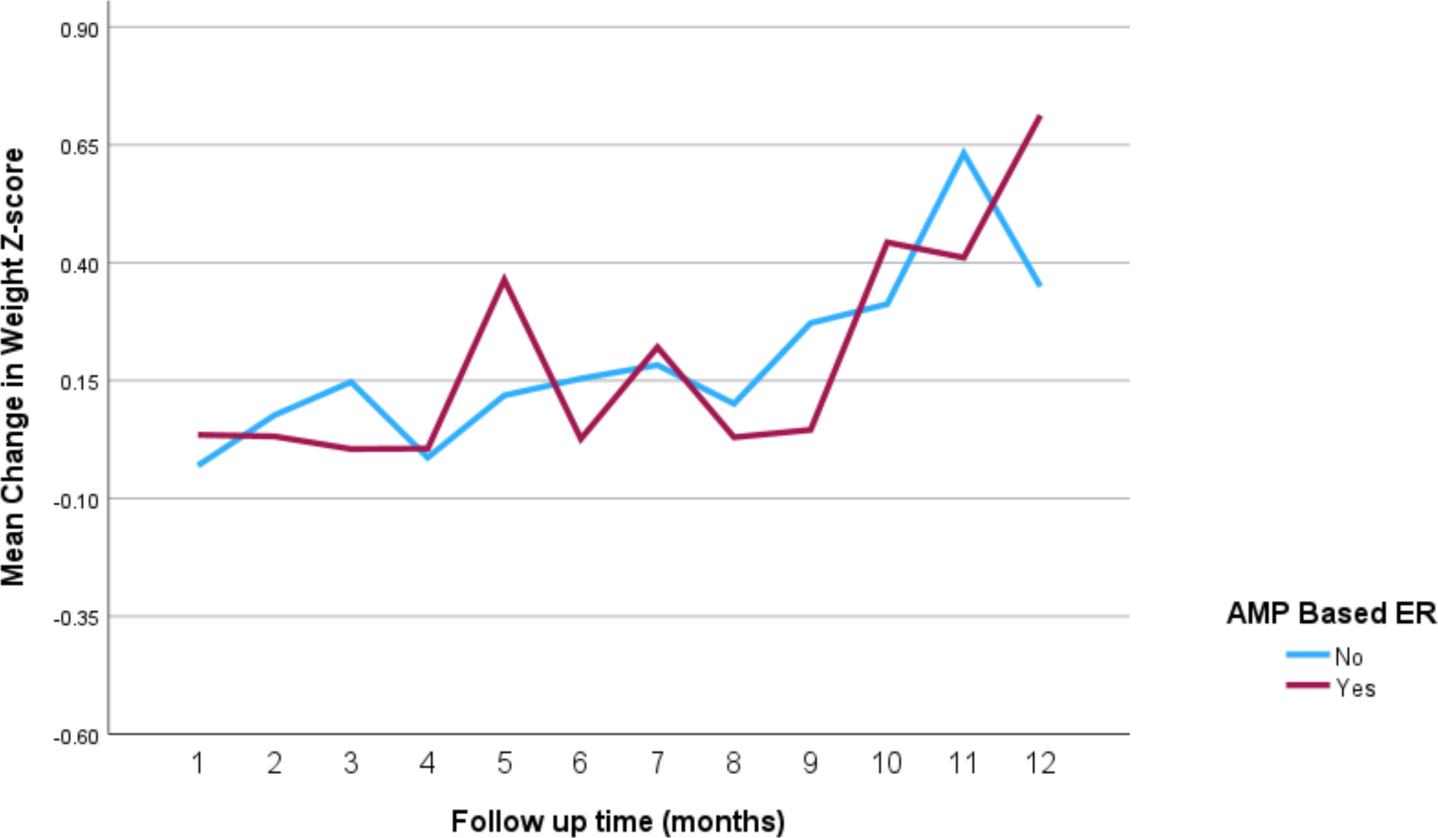
Impact of amphetamine-based extended release (Lisdexamfetamine or amphetamine mixed salts extended release) on weight over a 12-month period. AMP-based ER, amphetamine-based extended release (lisdexamfetamine or amphetamine mixed salts extended release).

The impact of the different stimulant medications on weight-for-age *z*-scores over the 12-month follow-up period is illustrated in **Figure 4** through **7**. Patients who started on methylphenidate immediate release (**Figure 4**) showed relatively stable weight-for-age *z*-scores, with a slight decrease around the fourth month, followed by a gradual increase. Similarly, methylphenidate OROS (**Figure 5**) also led to a decrease in *z*-scores around the fourth month, but the scores began to increase thereafter. For those on amphetamine mixed salts immediate release (**Figure 6**), there was a more pronounced decline in weight-for-age z-scores during the early months, followed by a sharp increase after the tenth month. Patients treated with amphetamine-based extended-release medications (**Figure 7**) experienced initial fluctuations in their *z*-scores, with a significant increase observed by the 12th month.

The analysis explored the impact of stimulant medications on weight-for-age *z*-scores in patients with ADHD and co-morbid conditions such as ASD and sleep problems. As shown in **Table 3**, while the presence of ASD itself did not have a statistically significant impact on weight changes (*p* = 0.605), there was a significant positive change in weight-for-age z-scores over time (*p* = 0.019), indicating that, regardless of ASD, patients generally experienced an improvement in their weight relative to age and sex during the follow-up period. However, the interaction between ASD and time was not significant (*p* = 0.850), suggesting that ASD did not modify the effect of time on weight changes. Furthermore, **Table 4** shows that the presence of sleep problems did not significantly affect weight changes (*p* = 0.138), although time continued to be a significant factor in weight change (*p* < 0.001).

**Table 3:** Impact of stimulant medications on weight *z*-score in patients with ADHD and co-morbid ASD.

Parameter	Regression coefficient	95% Wald confidence interval		
		Lower	Upper	*P*-value
(Intercept)	−1.201	−1.619	−0.782	< 0.001
ASD	0.001	−0.002	0.004	0.605
Time in months	0.067	0.011	0.124	0.019
ASD * Time	0.0000288	0.000	0.000	0.850

ASD, autism spectrum disorder.

**Table 4:** Impact of stimulant medications on weight *z*-score in patients with ADHD and co-occurring sleep problems.

Parameter	Regression coefficient	95% Wald confidence interval		
		Lower	Upper	*P*-value
(Intercept)	−1.089	−1.538	−0.640	< 0.001
Sleep problem	−0.001	−0.002	0.000	0.138
Time in months	0.031	0.018	0.044	< 0.001
Sleep problem * Time	0.0000564	0.000043	0.00006906	< 0.001

## 4 Discussion

The main aim of this study was to evaluate the effect of psychostimulant medications on the weight in children and adolescents diagnosed with ADHD. The results offered insights into this topic, allowing a detailed comparison with existing literature and the opportunity to correlate global data with that seen in the UAE.

The most common ADHD presentation noted in our patient population was the combined type (45.8%), who are more likely to be referred to clinical services as per a previous meta-analytic review (27). Additionally, the most frequently prescribed medication was found to be MPH immediate release followed by MPH OROS, Amphetamine-based extended release and AMP immediate release. Methylphenidate being the most commonly prescribed medication among the ADHD patient population aligns well with the literature. In a global observational analysis of ADHD medication published in 2018, population-based database analysis revealed that methylphenidate was the most commonly prescribed medication in most countries (28). Another study by Morkem et al. published in 2020 analyzed the trends of ADHD medications in Canadian Primary Care and found that methylphenidate accounted for 65.0% of all prescribed medications among patients with ADHD (29). The common use of methylphenidate has been attributed to its favorable effects on attenuating core symptoms of ADHD, including impulsivity, hyperactivity, and inattention while having a lower risk of serious adverse side effects (30).

Throughout the treatment of patients with ADHD in this study, the mean weight-for-age *z*-scores initially showed a slight decrease, particularly around the fourth month, but generally stabilized and partially recovered over the 12-month period. The stabilization of weight-for-age *z*-scores suggests that, while stimulant medications may initially impact weight, their long-term effects may be less pronounced, with patients eventually returning to expected growth trajectories. A global literature review published in 2017 assessed the effects of ADHD medications on weight and appetite and found a similar pattern to our study; in the first 3–6 months, a pattern of weight loss was observed, but growth patterns and charts were expected and predicted to rejoin the growth pattern of patients who were not on any medications (31). One of the proposed mechanisms for this weight loss was a dysregulation of appetite associated with intracerebral activation of the insular lobe leading to a sensation of disgust while on methylphenidate (31).

Another review published in 2008 by Faraone et al. analyzed the effects of stimulant medications such as methylphenidate and found an overall pattern of weight loss, rather than weight gain, while on these medications (32). However, as in our study, it was seen that the effect of stimulant medication on weight attenuates over time and it was interpreted in the review that these effects were likely dose dependent and would not impact the overall growth and development into adulthood (32).

Overall, the pattern seen in the patients in this study correlates well with the existing literature, and this could be further confirmed and analyzed with a larger sample size.

Another important factor analyzed in this study was the effect of age and gender on the pattern of weight change in patients with ADHD on medication. The longitudinal analysis revealed a significant positive association between age and weight-for-age *z*-scores, suggesting that as patients aged, their weight relative to expected growth patterns improved. Gender did not have a statistically significant impact on weight changes, indicating similar effects of stimulant medications on weight for both male and female patients. The limited sample size of females in this study precludes definitive conclusions regarding gender differences in weight changes, though the overall findings suggest no significant gender impact on the effects of stimulant medications. The likely reason for this is that the female population with ADHD (19.8%) in this study was much less than the male population (80.2%), which makes it difficult to identify a significant pattern. However, the significant difference in the ratio of males to females is consistent with a previous study conducted on a sample of 428 children and adolescents in the UAE (33). This discrepancy can be attributed to many factors including the higher diagnosed ADHD cases in males compared to females, which is approximately 4:1. Also, the fact that boys are more likely to be referred and prescribed psychostimulants due to their disruptive behavior. A larger sample size would likely minimize the impact of this issue and may be considered for a future study.

When comparing our findings with existing research, it is relevant to discuss a study conducted in Florida, USA, by Gurka et al. They aimed to assess how numerous classes of psychotropic medications, including stimulants, antipsychotics, and antidepressants, affect BMI trajectories of individuals with ADHD. Their results indicated that the impact on weight was more noticeable in younger age groups across all medication classes (34), aligning with our study findings. It is important to note that since our study focused on stimulant medications, a direct comparison cannot be made regarding other medication classes prescribed to children with ADHD.

GEE for longitudinal data analysis showed a significant positive association between age and weight-for-age *z*-scores (*p* < 0.001), indicating that as patients aged, their weight relative to expected growth patterns improved. Similarly, time was significantly associated with improvements in weight-for-age *z*-scores over the follow-up period (*p* < 0.001). This represents the decreased probability of ADHD medication impacting the overall growth of a child or adolescent. However, it is important to note that, while the literature has typically indicated that it is unlikely for ADHD medications to impact patient growth and development into adulthood, it must be noted that the data for patients in this study was assessed over 12 months while they were taking medications and therefore cannot account for effects of medication on weight beyond the 12-month period over which the patients were assessed.

It is well documented that ADHD is associated with multiple comorbidities such as ASD, tic disorders, and learning disorders (33, 35). This was not different in our patient population, where numerous comorbidities were observed in the patient sample. For instance, ASD was observed in 38.8% and learning disorder in 25.7% which correlates with known findings. However, the incidences of some conditions differed from those seen in the literature review. For example, tic disorders were only identified in 5.3% of patients in this sample, whereas it is quite commonly associated with ADHD in the literature. This difference may have been caused by an undiagnosed existing tic disorder or no formal diagnosis being present. Other possible reasons for discrepancies may be attributed to sample size, the use of a single center, and/or population differences.

The analysis found that the presence of ASD did not have a statistically significant impact on weight changes (*p* = 0.605), although time remained a significant factor in weight improvement, indicating that patients generally experienced an improvement in their weight-for-age *z*-scores over the follow-up period. Previous studies highlighted the neurosenstivity of children with autism, which makes them more prone to side effects of psychostimulants (36), hence more appetite suppression and less weight gain. In fact, children with ASD are either at greater risk or have the same risk of obesity compared to the other normally developing peers (37, 38). Our study excluded children who are taking medications that causes weight gain. Risperidone, a commonly prescribed medication for irritability or aggression among individuals with autism (39), is known for causing weight gain and most evidently in children (40).

Although it is known that sleep disorders can result in weight gain (41), this was not found in our study as there was no significant impact of having a sleep problem on weight measurements. As having a sleep problem refers to some difficulties in sleep, not a formal diagnosis of a sleep disorder. It is likely this is why the same finding was not observed in our study.

In the context of the Middle East, it is essential to consider potential biases arising from gender differences and cultural practices. Males and females in this region often lead different lifestyles, which can impact the study outcomes. For example, there may be restrictions in eating habits for females, especially during adolescence, which can affect their nutritional status and weight (42).

While psychostimulant medications are generally accessible, adherence may vary due to cultural factors. In some cultures, there may be a stigma associated with taking psychiatric medication, which can affect adherence rates. Parents may be reluctant to medicate their children due to concerns about social judgment or potential long-term effects, despite the availability of these medications. Furthermore, traditional beliefs can influence help-seeking practices, impacting adherence to prescribed medication regimens (43). Some patients might refuse to use psychotropic medications and rather seek traditional medicine, such as visiting faith healers or applying blessed olive oil (44). This practice can lead to a decrease in the number of children with ADHD taking psychostimulants. However, the impact of this factor is limited due to the characteristic of the represented sample, which is diverse with children from different nationalities, ethnicities, and religions, reflecting the characteristics of the UAE population.

We acknowledge that the study did not control for cultural attitudes towards treatment, which could affect the results. Future studies should consider these factors to provide a more comprehensive understanding of the impact of psychostimulant medications in diverse cultural settings.

### 4.1 Strengths, limitations, and generalizability

This study demonstrates several strengths. First, its pioneering nature as the first study of its kind in the region underscores its significance, filling a crucial gap in the existing literature. The study’s comprehensive design, spanning a minimum period of 6 months, allows for a thorough examination of the long-term effects of psychostimulant medications on weight, providing valuable insights into both short-term and sustained outcomes. The well-defined inclusion criteria and meticulous data collection procedures, enhance the study’s internal validity. Furthermore, the inclusion of a diverse sample representative of the UAE’s population ensures the generalizability of findings to a broader context.

In terms of limitations, there were missing data in most of the studied patients especially their weight which is supposed to be followed for 12 months after the initiation of the psychostimulant medication. However, there are many reasons for this including the quarantine during COVID pandemic, as weight is usually measured during the visit to the hospital. Also, the lack of regular monthly follow-ups in most of the patients.

Another notable limitation of this study is the unavailability of consistent height measurements during follow-up visits. The absence of this data impeded our ability to calculate body mass index (BMI) percentiles adjusted for age and sex, which are considered a more comprehensive indicator of growth and nutritional status in pediatric populations (45). Consequently, we relied on weight-for-age *z*-scores to standardize and analyze weight changes over time. While weight-for-age *z*-scores provide valuable insights, they do not account for variations in height growth, potentially limiting the depth of our analysis regarding the effects of psychostimulant medications on overall growth patterns. Future studies should aim to incorporate regular and systematic collection of both weight and height measurements to enable a more thorough assessment using BMI percentiles, thereby enhancing the accuracy and applicability of the findings.

Another limitation of this study is that the population consists of children referred to a specialty clinic. This referral pattern may result in a higher prevalence of certain conditions, such as ASD, which was observed in 38% of our sample. This percentage likely exceeds what would be found in a more community-oriented sample, potentially limiting the generalizability of our findings.

### 4.2 Implications and areas for future research

Several avenues for future research are apparent. First, the observed patterns of weight change over the 12-month period raise questions about the long-term effects beyond this timeframe. Further investigations with extended follow-up periods could provide a more comprehensive understanding of the sustained impact of psychostimulants on weight in this population. Additionally, the study highlights the influence of age and gender on weight changes, suggesting the need for more nuanced examinations within specific demographic groups. A larger sample size could enable a more robust analysis of gender-specific trends and age-related variations. Furthermore, the presence of comorbidities warrants further exploration. Future research should delve into the complex interactions between psychostimulant medications, neurosensitivity, and comorbid conditions, considering factors like medication type and dosage. Addressing these aspects will contribute to a more nuanced understanding of the multifaceted effects of ADHD medications, guiding clinicians in tailored treatment approaches.

## 5 Conclusion

In conclusion, this study provides valuable insights into the effects of psychostimulant medications on the weight of children and adolescents diagnosed with ADHD. The predominant prescription of methylphenidate aligns with global trends and the observed patterns of initial weight loss followed by subsequent gain mirror existing literature. The influence of age, gender, and comorbidities on weight changes adds complexity to the picture, emphasizing the need for personalized treatment approaches. Despite certain discrepancies from expected outcomes, the study’s strengths, including its pioneering nature and comprehensive design, contribute to the growing body of knowledge in this field. Nevertheless, the limitations, such as missing data and the relatively short follow-up period, highlight the challenges in conducting longitudinal research. Future studies addressing these limitations and exploring the intricacies of medication effects on specific subgroups will enhance our understanding and guide clinical decision making in the treatment of ADHD in pediatric populations.

## Data Availability

The original contributions presented in the study are included in the article/supplementary material, further inquiries can be directed to the corresponding author.
